# Characterization of MicroRNA expression profiles in the ovarian tissue of goats during the sexual maturity period

**DOI:** 10.1186/s13048-023-01318-8

**Published:** 2023-12-08

**Authors:** Yanyan Wang, Jianmin Wang, Qing Li, Rong Xuan, Yanfei Guo, Peipei He, Tianle Chao

**Affiliations:** 1 Shandong Provincial Key Laboratory of Animal Biotechnology and Disease Control and Prevention, College of Animal Science and Veterinary Medicine, Shandong Agricultural University, Tai’an, Shandong China; 2 Key Laboratory of Efficient Utilization of Non-Grain Feed Resources (Co-Construction By Ministry and Province), Ministry of Agriculture and Rural Affairs, College of Animal Science and Veterinary Medicine, Shandong Agricultural University, Tai’an, Shandong China

**Keywords:** Goats, Reproductive hormones, Sexual maturity, Ovary, MicroRNA

## Abstract

**Background:**

The ovary is an important reproductive organ in mammals, and its development directly affects the sexual maturity and reproductive capacity of individuals. MicroRNAs (miRNAs) are recognized as regulators of reproductive physiological processes in various animals and have been shown to regulate ovarian development through typical targeting and translational repression. However, little is known about the regulatory role of miRNAs in ovarian tissue development during sexual maturity in goats. To comprehensively profile the different physiological stages of sexual maturation in goats, we performed small-RNA sequencing of ovarian tissue samples collected at four specific time points (1 day after birth (D1), 2 months old (M2), 4 months old (M4), and 6 months old (M6)). In addition, we used ELISAs to measure serum levels of reproductive hormones to study their temporal changes.

**Results:**

The results showed that serum levels of gonadotropin-releasing hormone, follicle-stimulating hormone, luteinizing hormone, oestradiol, progesterone, oxytocin, and prolactin were lower in goats at the D1 stage than at the other three developmental stages (*P* < 0.05). The secretion patterns of these seven hormones show a similar trend, with hormone levels reaching their peaks at 4 months of age. A total of 667 miRNAs were detected in 20 libraries, and 254 differentially expressed miRNAs and 3 groups of miRNA clusters that had unique expression patterns were identified (|log2-fold change|> 1, FDR < 0.05) in the 6 comparison groups. RT‒qPCR was employed to confirm that the expression pattern of the 15 selected miRNAs was consistent with the Illumina sequencing results. Gene ontology analyses revealed significant enrichment of GO terms such as cell proliferation regulation, epithelial cell development, and amino acid transport, as well as important signaling pathways including the MAPK signaling pathway, the PI3K-Akt signaling pathway, and the oestrogen signaling pathway. Further miRNA‒mRNA regulation network analysis revealed that 8 differentially expressed miRNAs (chi-miR-1343, chi-miR-328-3p, chi-miR-877-3p, chi-miR-296-3p, chi-miR-128-5p, chi-miR-331-3p, chi-miR-342-5p and chi-miR-34a) have important regulatory roles in ovarian cell proliferation, hormone secretion and metabolism-related biological processes.

**Conclusions:**

Overall, our study investigated the changes in serum hormone and miRNA levels in the ovaries. These data provide a valuable resource for understanding the molecular regulatory mechanisms of miRNAs in ovarian tissue during the sexual maturity period in goats.

**Supplementary Information:**

The online version contains supplementary material available at 10.1186/s13048-023-01318-8.

## Introduction

Goats (*Capra hircus*) are an important type of domestic and commercial livestock in China and are an important source of meat, skin, fur and fibre for humans. However, the low fecundity of goats has seriously constrained the development of the goat industry [1]. In recent years, the Jining Grey goat has been used as an ideal model to study the growth and development process of sexually mature ovaries in domestic animals due to its precocious puberty, higher fertility and year-round oestrus [2]. Puberty (first ovulation) occurs at 2 months of age in Jining Grey goats, and sexual maturity occurs significantly earlier (3–4 months) in these goats than in other goats [3, 4]. This suggests that key reproductive traits, such as ovarian function and hormone regulation, are manifested in Jining Grey goats in the early growth stages. Their excellent reproductive characteristics provide more possibilities for the study of sexual maturity.

Sexual maturation refers to the period from birth to full sexual maturity (capable of normal reproduction), and past studies have reported that sexual maturity is directly related to the maturation of the hypothalamic–pituitary–ovarian (HPO) axis. The ovary plays a crucial role during sexual maturation by directly mediating the secretion of oestrogen and progesterone and maintaining reproductive capability through follicular development and ovulation [5]. With the onset of sexual maturity, the endocrine function of the animal changes, and ovarian tissues show different physiological states and functional manifestations. For example, the HPO axis is activated at the onset of puberty. The levels of gonadotropin-releasing hormone released by the hypothalamus and gonadotropins (follicle-stimulating hormone and luteinizing hormone) secreted by the pituitary gland change. This results in the development of the ovaries of the goat, the secretion of hormones such as oestrogen and progesterone, and ultimately, oestrous and ovulation [6]. During sexual maturation, follicles develop rapidly, ovulation frequency increases, and granulosa cells secrete more oestrogen and progesterone. During this stage, the ovaries are most active in terms of their reproductive and endocrine functions to support the mature oestrous cycles and normal ovulation [2]. Therefore, sexual maturation is the main stage of ovarian development. However, the biological mechanisms underlying the developmental changes in ovarian tissue during sexual maturation are still poorly understood.

MicroRNAs (miRNAs) are single-stranded and noncoding RNAs that are key posttranscriptional regulators. In animals, miRNAs control gene expression by interacting with the 3’ untranslated region (3’UTR) of the target mRNA and then degrading or inhibiting mRNA translation [7]. These molecules play a role in a variety of biological processes, including cell proliferation [8], cell differentiation [9], apoptosis [10], tumorigenesis [11], hormone secretion [12], and metabolism [13]. There is growing evidence that miRNAs play a widely recognized role in almost all ovarian biological processes, including ovary maturation, ovarian cell development, luteal development and regression [14–16]. For example, miR-17-5p regulates the expression of the antiangiogenic factor tissue inhibitor of metalloproteinase 1, which is involved in corpus luteum angiogenesis, the lack of which can lead to infertility [17]. miR-34a regulates the proliferation and function of luteal cells during the transition of the corpus luteum from the developmental to the functional stages [18]. There is a correlation and temporal relationship between the overexpression of miR-21 and the posttranscriptional regulation of programmed cell death 4 (PDCD4) during oocyte maturation [19]. Let-7 family members play critical roles in cell fate determination and have been implicated in the regulation of housekeeping genes during ovarian development [20]. Several studies have shown that miR-133 family members are involved in the regulation of a variety of cellular processes, including cell proliferation, migration, and invasion [21]. miR-133b binds to the FOXL2-3’UTR in epithelial-like granulosa cells (GC) and reduces the expression of FOXL2. Reduced binding of FOXL2 to the promoters of STAR and CYP19A1 promotes their expression, thereby promoting oestrogen secretion by GCs [22]. Therefore, studying the expression patterns and potential functions of miRNAs can help us better understand the molecular regulatory mechanisms of ovarian development during sexual maturation.

In the present study, we measured serum levels of reproductive hormones to study their temporal changes at four specific time points (1 day after birth (D1), 2 months after birth (M2), 4 months after birth (M4), and 6 months after birth (M6)) in Jining Grey goats and carried out small-RNA sequencing of ovarian tissue samples to detect the differentially expressed miRNAs and their potential functions during sexual maturation in goats. These results can help to better understand the molecular regulatory mechanisms of ovarian development during the biological process and provide an effective basis for further exploring the improvement of the reproductive regulatory network of the Jining Grey goat, thus promoting progress in the field of goat breeding.

## Materials and methods

### Ethics approval and consent to participate

Animal experiments were conducted with the consent and guidance of the Animal Care and Use Committee of Shandong Agricultural University (SDAUA-2023–157). All personnel involved in the experiment were trained and strictly followed the experimental procedures.

### Experimental animals and tissue sample collection

In this study, 20 healthy female Jining Grey goats were selected from the Jining Grey Goat Breeding Farm in Jiaxiang, Shandong Province (Jining, China) (Supplementary Table 1). These goats were categorized into four age groups: D1 (2.60 ± 1.52 days old), M2 (2.07 ± 0.04 months old), M4 (4.05 ± 0.05 months old), and M6 (6.06 ± 0.06 months old), with five goats in each age group. All goats had free access to food and were bred and managed under identical conditions. All goats were slaughtered uniformly at their respective cut-off dates, and ovarian tissues were collected for transcriptome analysis. Finally, the collected samples were snap-frozen in liquid nitrogen and then stored in a freezer at − 80 °C until sequencing analysis. To differentiate the samples, ovary samples from 1-day-old goats were labelled D1-1, D1-2, D1-3, D1-4 and D1-5; ovarian samples from 2-month-old goats were labelled M2-1, M2-2, M2-3, M2-4 and M2-5; ovarian samples from 4-month-old goats were labelled M4-1, M4-2, M4-3, M4-4 and M4-5; and ovarian samples from 6-month-old goats were labelled M6-1, M6-2, M6-3, M6-4 and M6-5.

### Blood sample collection and determination of hormone concentrations

Blood (10 mL) was collected from the jugular vein of the experimental goats, centrifuged at 3000 rpm for 10 min and then stored at 4 °C. Serum was separated and stored at − 20 °C for determination of hormone concentrations. Hormone levels were measured by enzyme-linked immunosorbent assay (ELISA) [23]. The following ELISA kits purchased from Qingdao Mdbio Biotech Co., Ltd. (Qingdao, Shandong, China), for goat follicle-stimulating hormone (FSH), goat luteinizing hormone (LH), goat oestradiol (E2), goat progesterone (PROG), goat oxytocin (OT) and goat prolactin (PRL), were used to measure FSH, LH, E2, PROG, OT and PRL, respectively. All assays were repeated three times. The experimental procedures were carried out according to the instructions provided in the kits.

### RNA isolation, small RNA library construction and sequencing

Total RNA was extracted from 20 ovarian tissue samples with TRIzol RNA extraction reagent (Invitrogen, Carlsbad, CA, USA) according to the manufacturer's instructions. The RNA concentration, integrity, and purity were determined using a NanoDrop 2000 spectrophotometer (Thermo Scientific, Wilmington, DE, USA) or an Agilent Bioanalyzer (Agilent, Santa Clara, CA). An RNA integrity number (RIN) of > 8.0 was used as the criterion for Illumina sequencing. Total RNA was isolated from ovarian tissue, and 20 small RNA libraries were established using the NEB Next® Multiplex Small RNA Library preparation kit (NEB E7300L). Briefly, 1 μg of total RNA was taken from each small RNA library and electrophoresed on a 12% PAGE gel (in TBE buffer) (Invitrogen) to isolate small RNA fragments ranging from 18 to 30 nt in length. The above small RNAs were used as a template, and the 3’ and 5’ adaptors were ligated to the 3’ and 5’ ends of the small RNA. First-strand cDNA was synthesized by reverse transcription with specific primers. After PCR amplification, the 140–160 bp sequence was purified, and a miRNA sequencing library was constructed. The libraries were subjected to sequencing on Illumina platforms with the SE50 (single-end 50 bp, SE50) strategy after quality tests. All sequencing was outsourced to Novogene Bioinformatics Technology Co., Ltd. (Beijing, China).

### Sequencing data preprocessing and comparison

Based on the quality assessment results [24], the sequencing adapters were processed with Cutadapt software to exclude from the raw data reads containing poly-N, reads with 5’ adapter contaminants, reads without 3’ adapter or the insert tag, reads containing poly-A, T, G or C, reads with 10% or more unknown bases and reads greater than 30 bp or less than 18 bp in length and low-quality reads. Finally, clean reads with a sequencing quality (Q) greater than 30 were retained for further analysis. The filtered clean reads were subjected to sequence comparison with the RepeatMasker (http://repeatmasker.org/) and Rfam (http://rfam.xfam.org/) databases using Bowtie software [25]. Subsequently, the reads were annotated as repeat reads and noncoding RNAs (ncRNAs), such as rRNA, snoRNA, snRNA, and tRNA. Filtering for ncRNAs and repeat sequences yielded unannotated reads containing miRNAs. Filtered unannotated reads were aligned with the goat reference genome (ftp.ncbi.nlm.nih.gov/genomes/all/GCF/001/704/415/GCF_001704415.2_ARS1.2) to determine the locus information on the reference genome. Several known miRNAs were identified, and novel miRNAs were predicted using miREvo [26] and miRDeep2 software [27] with mature and hairpin sequences from goat, cow and human in the miRBase 20.0 database [28]. miRDeep2 software computed transcripts per million (TPM) values for miRNA expression. The miRNAs were categorized into four groups based on the TPM values: high-expression group (TPM ≥ 500), medium-expression group (500 > TPM ≥ 10), low-expression group (10 > TPM ≥ 1), and ultralow-expression group (1 > TPM). The degree of variability of all miRNAs was examined by principal component analysis (PCA) with R. Subsequently, the detected miRNAs were subjected to family analysis to determine the conserved nature of the miRNAs throughout the evolutionary process based on their sequence similarities.

### Identification and screening differentially expressed miRNAs

Differential expression analysis was performed using DESeq2 software [29]. We treated the samples of the same age group as duplicates and compared samples of different ages (i.e., D1 vs. M2, D1 vs. M4, D1 vs. M6, M2 vs. M4, M2 vs. M6 and M4 vs. M6). miRNAs were considered to be differentially expressed when |log2FC|> 1 and FDR < 0.05. The total amount of differentially expressed miRNAs between different comparison groups was calculated using the R package UpSetR [30]. Heatmaps were plotted using the R package pheatmap to show the differentially expressed miRNAs. Volcano maps of the four groups of differentially expressed miRNAs were also plotted. The top 20 differentially expressed miRNAs at different developmental stages were then filtered out and displayed in bar charts.

### Temporal pattern analysis of miRNAs

To further characterize the expression pattern of differentially expressed miRNAs, we used Mfuzz software [31] to cluster differentially expressed miRNAs using the c-means method and plotted the expression levels of DE miRNAs in ovarian tissue at the four different developmental stages in a line graph.

### Target gene prediction and functional enrichment analysis of miRNAs

Both RNAhybrid [32] and miRanda software [33] were used to predict the target genes of differentially expressed miRNAs. The overlapping results of the two datasets were used as the final miRNA targets.

All differentially expressed genes were subjected to GO functional annotation and KEGG pathway enrichment analysis using the online software DAVID [34]. All goat annotated genes were used as the background list from which target genes were selected. *P* values were determined by the hypergeometric distribution test and corrected by Benjamini‒Hochberg multiple testing to derive the FDR, and the gene term or pathway was considered to be significantly enriched when the FDR was < 0.05. Genes related to ovarian development, cell growth, and hormone and substance metabolism were screened based on the results of gene function analysis.

### miRNA and target gene regulatory network construction

Potential target genes and corresponding miRNAs associated with ovarian development were screened based on the results of GO and KEGG analysis. miRNAs and target genes were mapped to the regulatory network of differentially expressed miRNAs and target genes using Cytoscape [35]. The hub miRNAs and hub genes were screened under the condition of degree score ≥ 7.

### Validation of quantitative real-time PCR (qRT‒PCR)

To validate the RNA-Seq results, 15 differentially expressed miRNAs were randomly selected from the transcriptome sequencing data and confirmed by quantitative real-time RT‒PCR. miRNA primers were designed by miRprimer software (see Supplementary Table 2) [36]. miRNA quantification was performed according to the instructions of the SYBR Green Premix Pro Taq HS qPCR Kit II (Code: AG11702) and the PCR amplification was performed on a LightCycler 480 instrument (Roche). The specific reaction conditions were as follows: denaturation at 95 °C for 30 s, amplification with 40 cycles of 95 °C for 5 s and 60 °C for 30 s and melting curve construction with steps of 95 °C for 60 s, 55 °C for 30 s, and 95 °C for 30 s. Expression levels were calculated using the 2(-Delta Delta Ct) method (2^−ΔΔCt^) using the U6 gene as an internal control (Supplementary Table 2) [37].

### Statistical analysis

Data analysis for this study was performed using R for statistical analysis, including one-way analysis of variance (ANOVA), least significant difference (LSD), and Tukey's multiple range test for multiple testing correction. The results were visualized using GraphPad 7.0 (GraphPad Software, USA).

## Results

### Measurement of serum levels of reproductive hormones

**Fig. 1:**
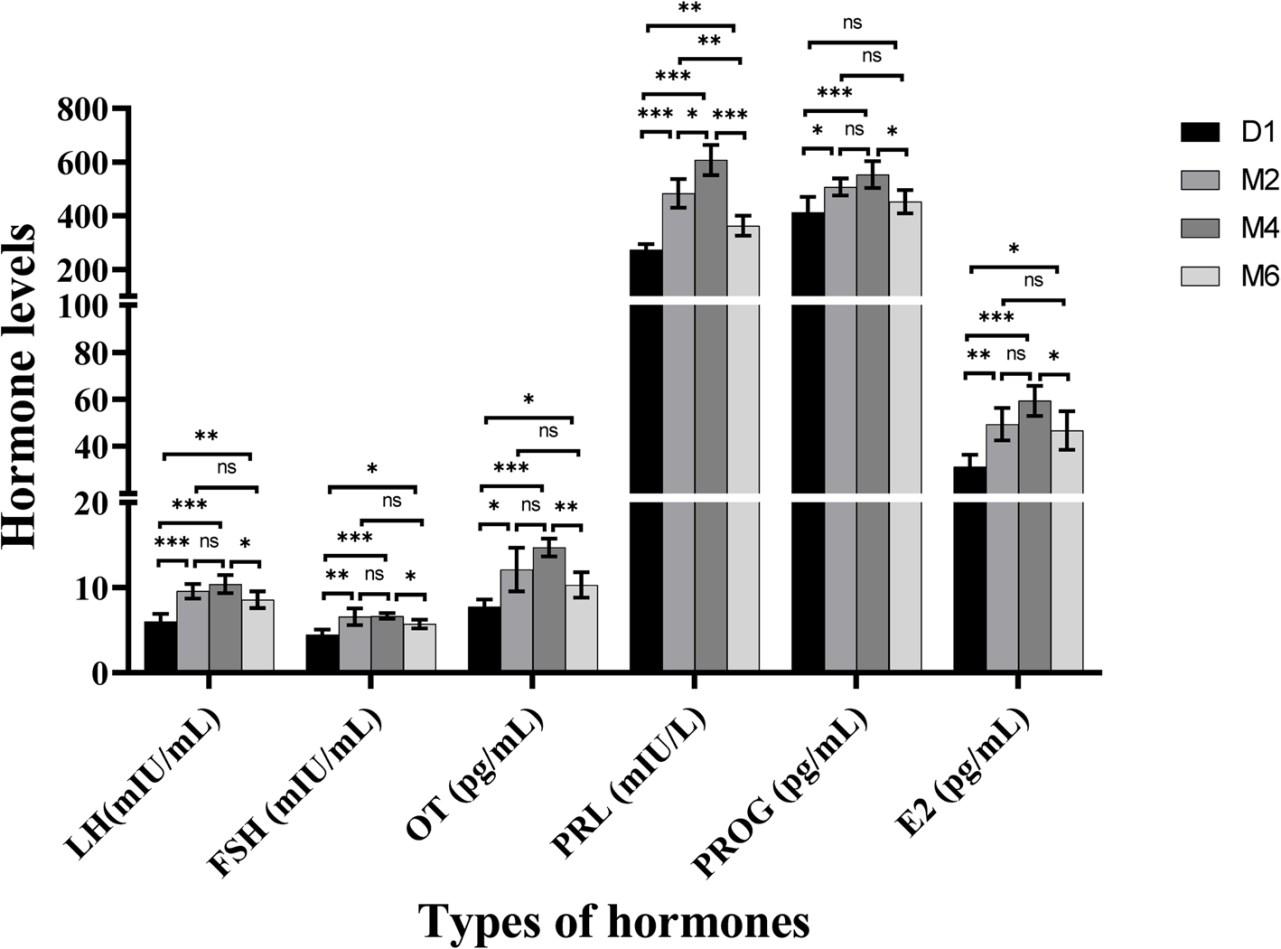
Levels of reproductive hormones in the serum of Jining Grey goats during the sexual maturity period. LH, luteinizing hormone; FSH, follicle stimulating hormone; OT, oxytocin; PRL, prolactin; PROG, progesterone; E2, estradiol; Values represent mean ± standard error. *p < 0.05, **p < 0.01, ***p < 0.001. n.s. = not statistically significant

There were significant differences in the serum levels of gonadotropin-releasing hormone, follicle-stimulating hormone, luteinizing hormone, oestradiol, progesterone, oxytocin and prolactin in goats at the D1 stage compared to the other three developmental stages (Fig. 1) (*P* < 0.05). The secretion patterns of these seven hormones showed a similar trend, with lower hormone levels at the D1 stage and peak levels at the M4 stage.

### Sequencing and alignment analysis of small RNAs

A small RNA library was constructed from the ovarian tissues of 20 Jining Grey goats, and we obtained 444001245 sequencing raw reads. These sequencing data were then quality controlled by filtering out 15905817 poor-quality reads and retaining 428095428 clean reads (Supplementary Table 3). The clean reads were compared with the RepeatMasker and Rfam databases to obtain the numbers of rRNA, tRNA, snoRNA, snRNA, and duplicate reads (Supplementary Table 4). Finally, 2093795 ± 168439, 1731920 ± 154660, 1508693 ± 87156, and 1862552 ± 171843 unannotated reads (mean ± SE) from Groups D1, M2, M4 and M6, respectively, were compared to the genome. Analysis revealed that more than 96% of reads were mapped to the genome (Supplementary Table 5).

### Identification of known and novel miRNAs

**Fig. 2:**
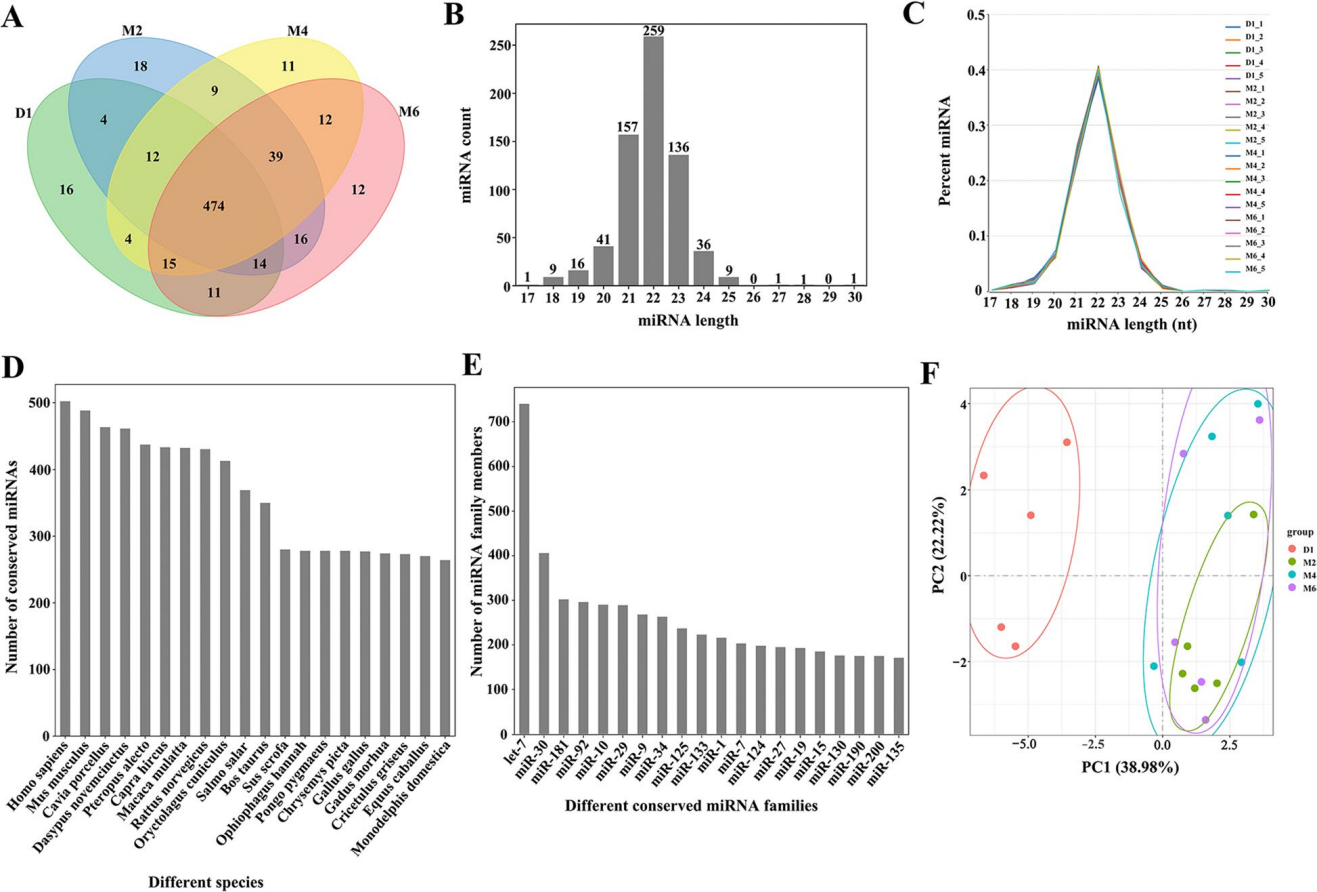
Identification and family analysis of known and novel miRNAs at different developmental stages. **A** Venn diagram of the number of miRNAs in ovary tissues at different developmental stages. **B** The number of miRNAs identified from the 20 libraries according to the length distribution. **C** Percentage distribution of identified miRNA lengths. **D** Statistics of the number of all miRNAs of certain species in the four stages. **E** Statistics on the number of miRNAs in different miRNA families. **F** Principal component analysis

A total of 667 miRNAs were detected in the 20 libraries; these included 422 known miRNAs and 245 newly predicted miRNAs, of which 474 miRNAs were expressed in all the libraries (Fig. 2A). As shown in Fig. 2B, C the lengths of all miRNAs were clustered between 20 and 24 nt, with the highest number of miRNAs (*n* = 259, 39% of all miRNAs) in the 22 nt group. To further determine the expression patterns of miRNAs in the four groups of ovarian tissues, the researchers classified the miRNAs into four groups according to the TPM values (Table 1). The number of miRNAs in the high-expression group was less in the D1 group than in the M2, M4 and M6 groups. In the medium- and low-expression groups, the number of miRNAs in the D1 group was higher than that in the other groups. In the ultralow-expression groups, the number of miRNAs in the M6 group was higher than that in the other groups.

**Table 1:** MiRNA expression levels of the four development stages

Different expression levels	D1	M2	M4	M6
Highly expressed miRNAs (TPM ≥ 500)	85	95	95	93
Mediumly expressed miRNAs (500 > TPM ≥ 10)	211	204	201	195
Lowly expressed miRNAs (10 > TPM ≥ 1)	146	126	127	128
Ultralowly expressed miRNAs (1 > TPM)	419	435	437	445

### miRNA family analysis

We compared miRNA sequence similarity across 134 species and identified 667 miRNAs out of 201 miRNA families, with let-7 being the most conserved (with matches in 116 species). Comparative analysis of the number of conserved miRNAs in different species showed that most miRNAs matched human miRNAs, with 502 miRNAs identified, followed by mouse miRNAs, with 488 miRNAs identified. We found 433 conserved miRNAs in goats (Fig. 2D). In addition, comparative analysis of the number of miRNAs in different miRNA families showed that the let-7 family had the highest number of miRNAs, with 740. Six miRNA families contained more than 100 miRNAs (Fig. 2E). Detailed results of the family analysis are shown in Supplementary Table 6.

### Identification of differentially expressed miRNAs

**Fig. 3:**
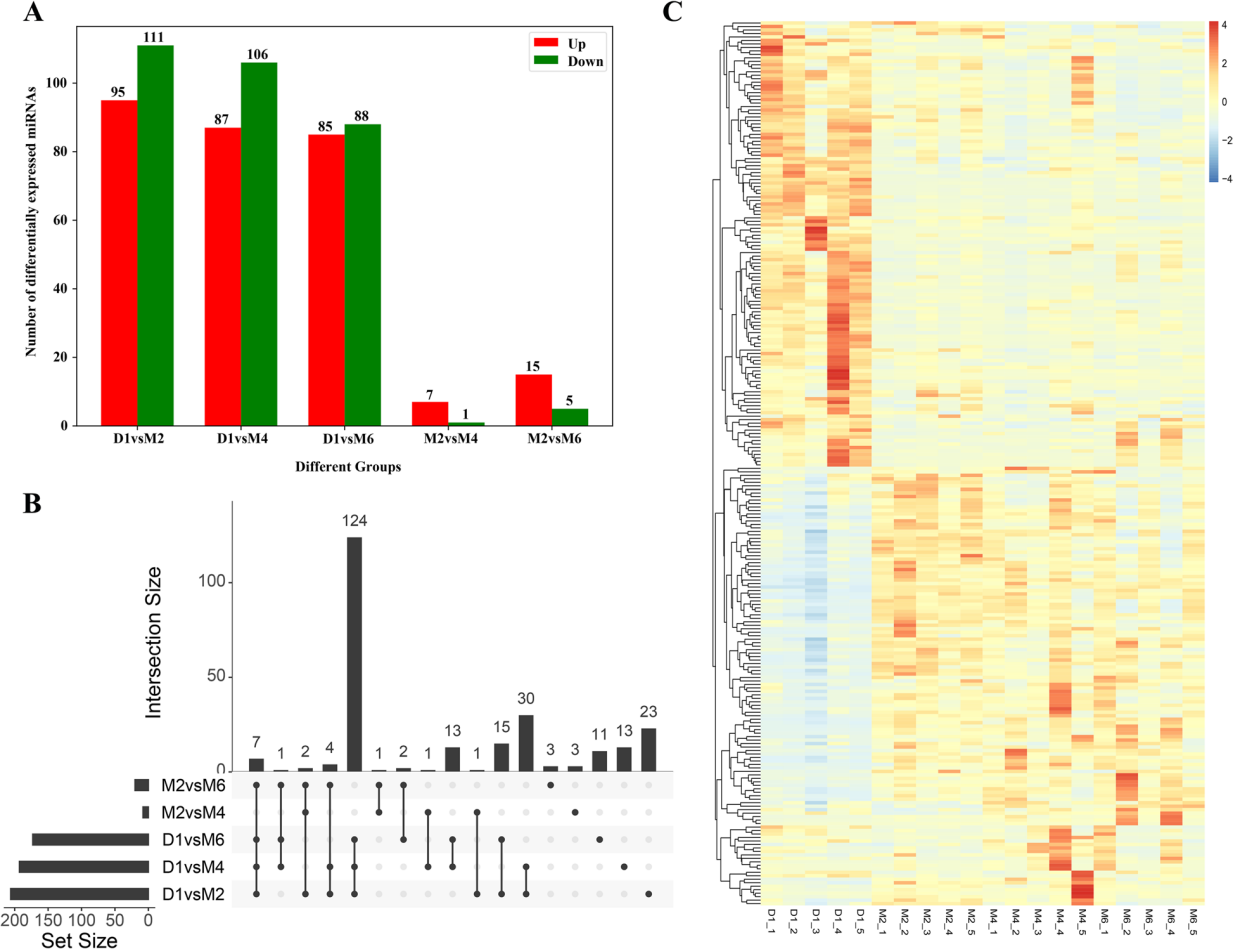
Identification of differentially expressed miRNAs. **A** Statistical results showing the number of up- and down-regulated miRNAs in different comparison groups. **B** The upset plot of the statistical analysis of the distribution of differentially expressed miRNAs in different comparison groups. **C** Heat map of all differentially expressed miRNAs

**Fig. 4:**
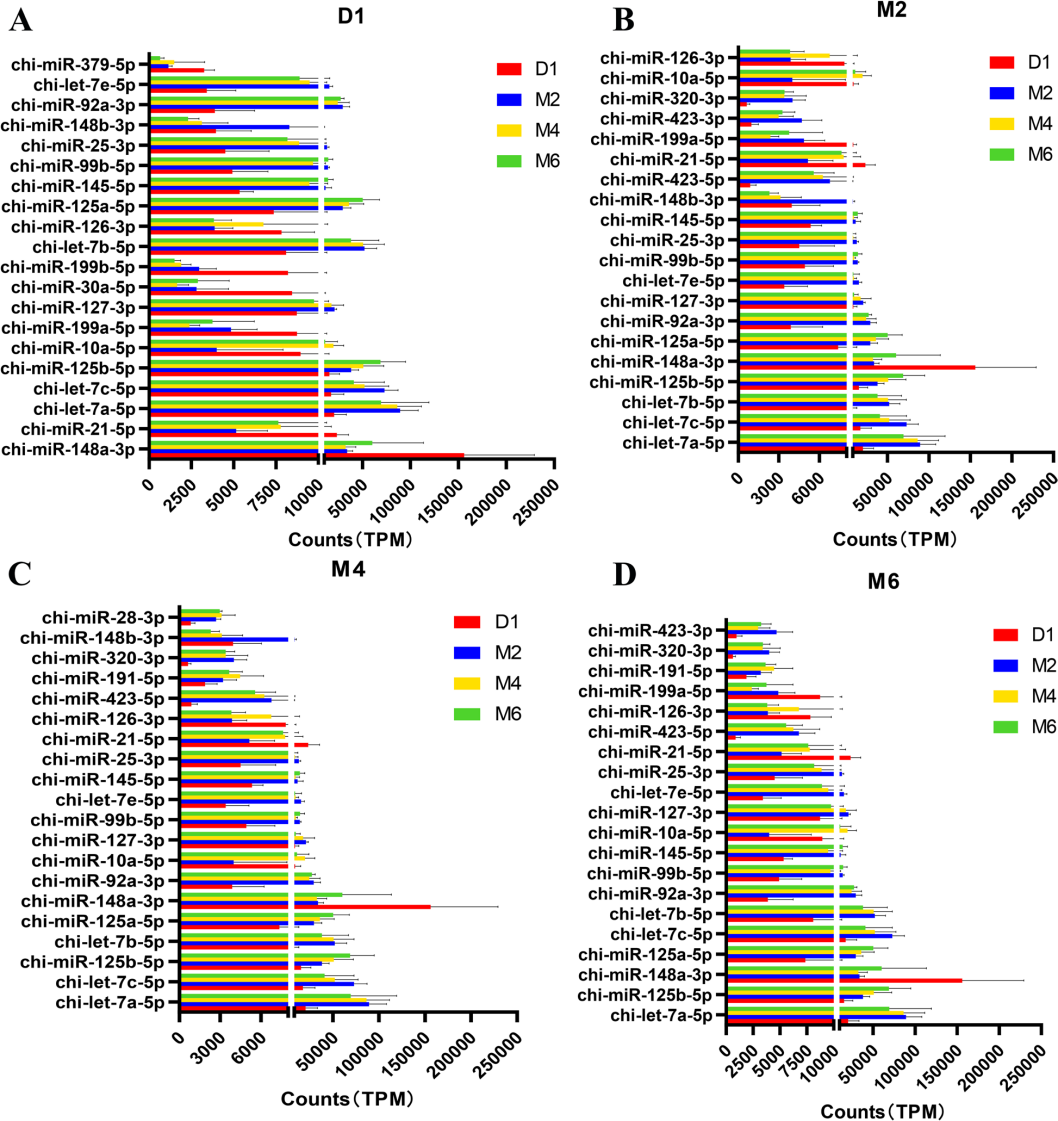
Expression patterns of the 20 most differentially expressed miRNAs at each developmental stage. **A** D1 stage; **B** M2 stage; **C** M4 stage; **D** M6 stage

Principal component analysis (PCA) of miRNA expression profiles of all samples revealed that samples at the same stage were clustered together (Fig. 2F). The normalized miRNA expression data were imported into DESeq2 software, and the differential expression hypotheses of D1 vs. M2, D1 vs. M4, D1 vs. M6, M2 vs. M4, M2 vs. M6 and M4 vs. M6 were tested by the Wald test and Benjamini‒Hochberg correction. Here, we identified 254 differentially expressed miRNAs in the six comparison groups (Fig. 3A). Among them, the highest number of differentially expressed miRNAs (95 upregulated miRNAs and 111 downregulated miRNAs) was found in the D1 vs. M2 comparison. Among them, miR-2332 was upregulated by 12.05-fold, and miR-136-5p was downregulated by 62.54-fold. Comparing D1 with M4, 87 miRNAs were upregulated, and 106 miRNAs were downregulated. It was found that novel_38 was upregulated by 27.10-fold, and miR-136-5p had the largest degree of downregulation, with a decrease of 144.59-fold. In the comparison between D1 and M6, 85 miRNAs were upregulated, and 88 miRNAs were downregulated. Let-7b-3p was the most upregulated (61.60-fold), while miR-376a was the most downregulated (108.43-fold). In the comparison of M2 with M4, 7 miRNAs were upregulated and 1 miRNA was downregulated, of which miR-429 was upregulated by 12.36-fold. In the comparison between M2 and M6, 15 miRNAs were upregulated, 5 miRNAs were downregulated, and miR-29b-3p was upregulated 10.17-fold. However, no differentially expressed miRNAs were found in the comparison of the M4 and M6 stages. Detailed differential expression results are shown in Supplementary Table 7. In addition, the UpSet plot showed the distribution of DE miRNAs between the different comparison groups (Fig. 3B), in which 124 miRNAs were differentially expressed between the D1 group and the M2 group, the D1 group and the M4 group and the M6 group, and 23 miRNAs were specifically differentially expressed in Group D1 versus Group M2. The heatmap showed that the expression of 254 DE miRNAs changed during the four periods (Fig. 3C). According to the expression levels of the 20 miRNAs with the greatest differences in expression at each developmental stage (Fig. 4), 15 miRNAs (chi-miR-148a-3p, chi-miR-21-5p, chi-let-7a-5p, chi-let-7c-5p, chi-miR-125b-5p, chi-miR-10a-5p, chi-miR-127-3p, chi-let-7b-5p, chi-miR-126-3p, chi-miR-125a-5p, chi-miR-145-5p, chi-miR-99b-5p, chi-miR-25-3p, chi-miR-92a-3p and chi-let-7e-5p) were expressed at high levels in ovarian tissues at four developmental stages (TPM ≥ 1000).

### Expression pattern analysis of differentially expressed miRNAs

**Fig. 5:**
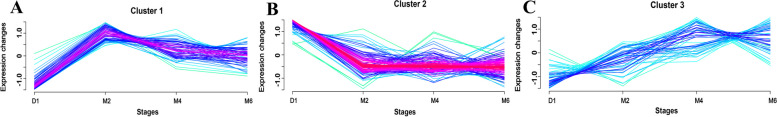
Expression pattern analysis of differentially expressed miRNAs. **A** Cluster 1 indicated a gradual increase in DE miRNAs from D1 to M2 stage, with the highest expression in group M2. **B** Cluster 2 indicated a gradual increase in DE miRNAs with the highest expression in D1 group. **C** Cluster 3 indicated a gradual increase in DE miRNAs from D1 to M4 stage, with the highest expression in group M4

Based on the cluster analysis of the expression levels of DE miRNAs in different periods, three clusters of DE miRNAs with different expression characteristics were obtained (Fig. 5 and Supplementary Table 8). The first cluster had 71 differentially expressed miRNAs, the second cluster had 132 differentially expressed miRNAs, and the third cluster had 51 differentially expressed miRNAs. This result reflects the stage specificity of miRNA expression in ovarian tissues.

### Prediction and enrichment analysis of the miRNA target genes

**Fig. 6:**
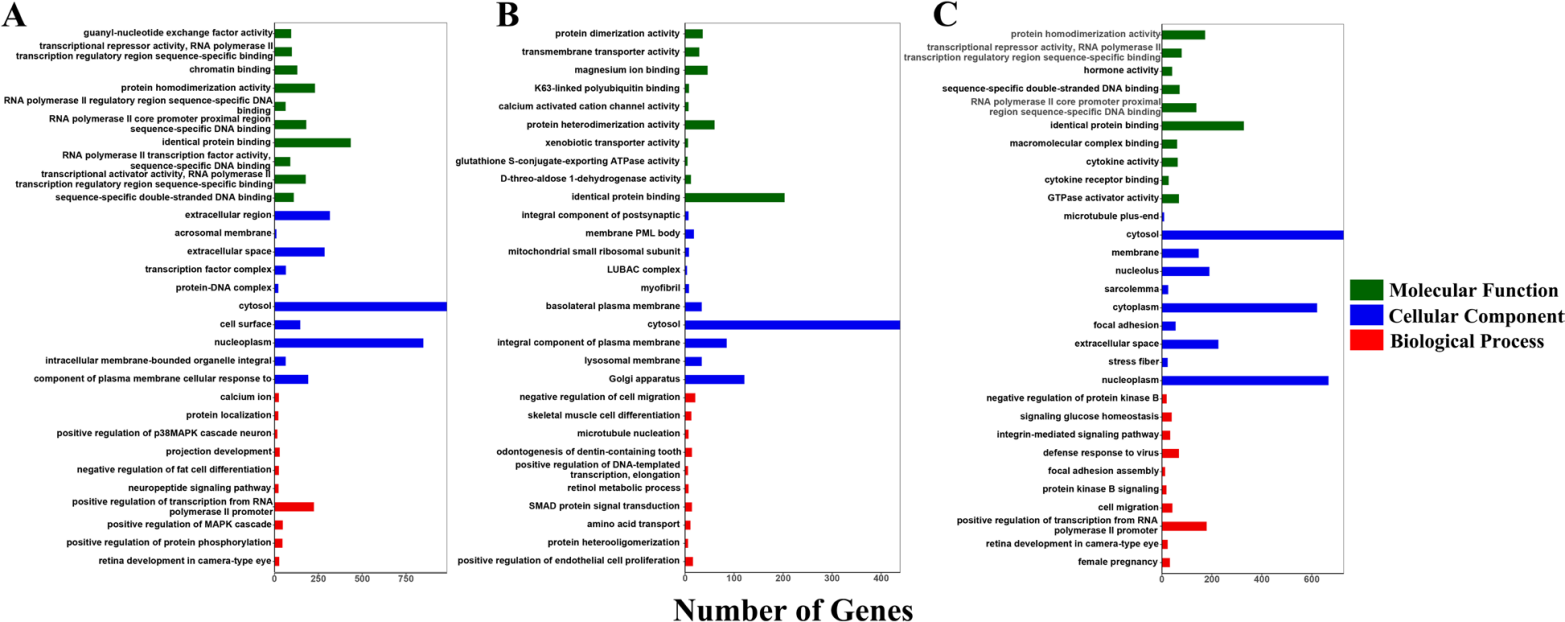
GO enrichment analysis of miRNA predicted target genes in three clusters. **A** cluster 1; **B** cluster 2; **C** cluster 3

**Fig. 7:**
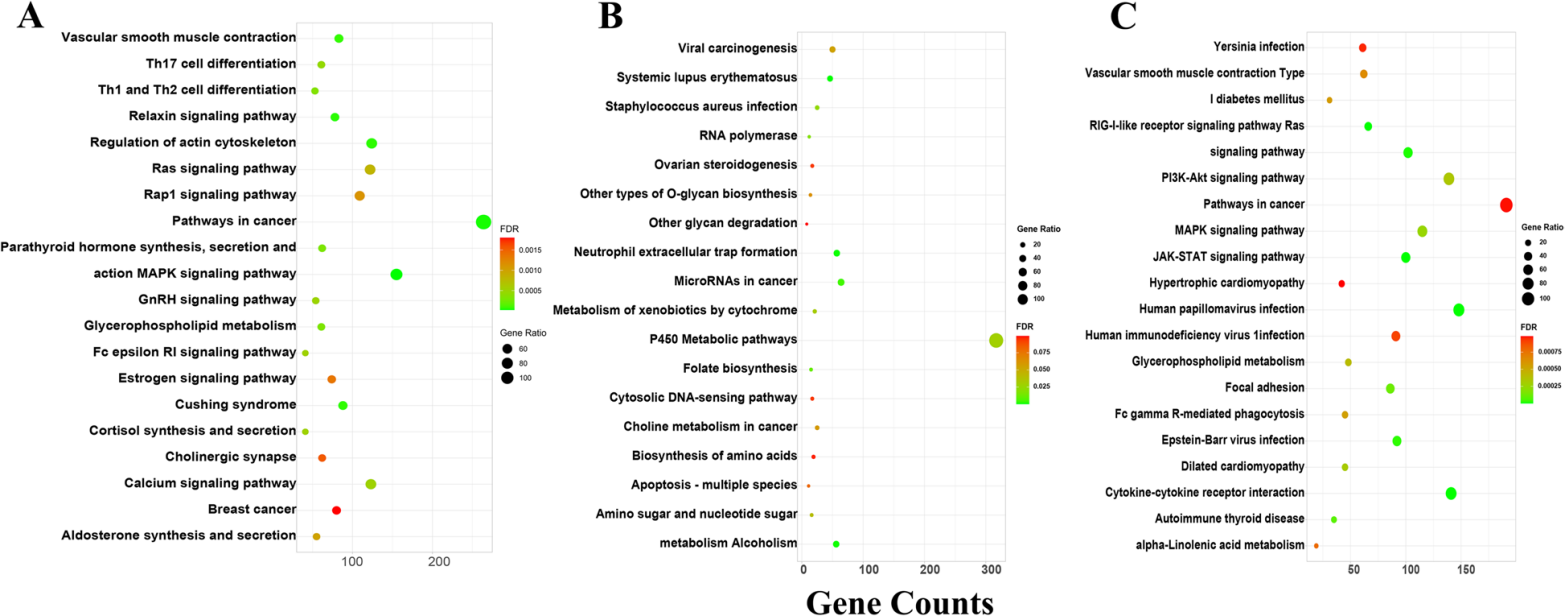
KEGG enrichment analysis of miRNA predicted target genes in three clusters. **A** cluster 1; **B** cluster 2; **C** cluster 3

The target genes of DE miRNAs were analysed by GO (Fig. 6) and KEGG pathway enrichment (Fig. 7) to clarify the main functions and regulatory pathways of DE miRNAs (Supplementary Table 9).

Cluster 1 showed the DE miRNAs that were highly expressed in the M2 group (Fig. 5A). The GO results identified 11,369 target genes, of which 4808 genes were enriched for 290 GO terms, including 57 cell component terms (CC), 53 molecular function terms (MF), and 180 biological process terms (BP). Three CC terms and eight MF terms were significantly enriched. The integral component of the plasma membrane (GO:0005887) and sequence-specific double-stranded DNA binding (GO:1990837) were the most significantly enriched CC and MF terms, respectively. In addition, terms related to cell proliferation and division, such as regulation of cell proliferation (GO:0042127), cell maturation (GO:0048469), and regulation of mitotic nuclear division (GO:0007088), as well as terms related to protein synthesis and metabolism, such as positive regulation of protein phosphorylation (GO:0001934), protein localization (GO:0008104), and phospholipid metabolic process (GO:0006644), were also enriched (Fig. 6A). The KEGG results showed that 2055 target genes were enriched in 96 pathways, of which 26 pathways reached significant enrichment, mainly including the MAPK signaling pathway, the Ras signaling pathway and the cAMP signaling pathway associated with ovarian development, as well as the oestrogen signaling pathway. (Fig. 7A). This may be closely related to ovarian development, first oestrous and ovulation.

Cluster 2 showed DE miRNAs that were highly expressed in Group D1 (Fig. 5B). The GO results identified 5814 target genes, of which 1494 genes were enriched in 132 GO terms, including 24, 34, and 74 in CC, MF, and BP terms, respectively. There were no significantly enriched terms. The top 30 enriched terms were mainly related to cell development, protein function regulation, and metabolism, such as epithelial cell development (GO:0002064), protein phosphorylation (GO:0006468), and retinol metabolic process (GO:0042572) (Fig. 6B). The KEGG results showed that 546 target genes were enriched in 18 pathways, and metabolic pathways, amino sugar and nucleotide sugar metabolism, nucleotide sugar metabolism, folate biosynthesis, and other signaling pathways related to cellular processes were enriched (Fig. 7B). This may be closely related to the early growth and development of ovarian tissue after birth.

Cluster 3 showed that the expression levels of DE miRNAs increased from D1 to M4 stage, with higher expression levels in the M4 group (Fig. 5C). The GO results identified 8443 target genes, of which 3805 genes were enriched in 271 GO terms, including 52, 58, and 161 CC, MF, and BP terms, respectively. The main enrichments were in the terms "female pregnancy" (GO:0007565), "cell migration" (GO:0016477), "defense response to virus" (GO:0051607), and "phosphatidylglycerol metabolic process" (GO:0046471) (Fig. 6C). The KEGG results showed that 1410 target genes were enriched in 105 pathways, of which 26 pathways were significantly enriched, including the RIG-I-like receptor signaling pathway, JAK-STAT signaling pathway, Toll-like receptor signaling pathway related to immunity, MAPK signaling pathway, PI3K-Akt signaling pathway, and Notch signaling pathway related to cell proliferation and ovarian development, as well as glycerophospholipid metabolism, alpha-linolenic acid metabolism, and fructose and mannose metabolism, which are related to metabolism (Fig. 7C).

### Regulatory networks of miRNAs and target genes associated with ovary development

**Fig. 8:**
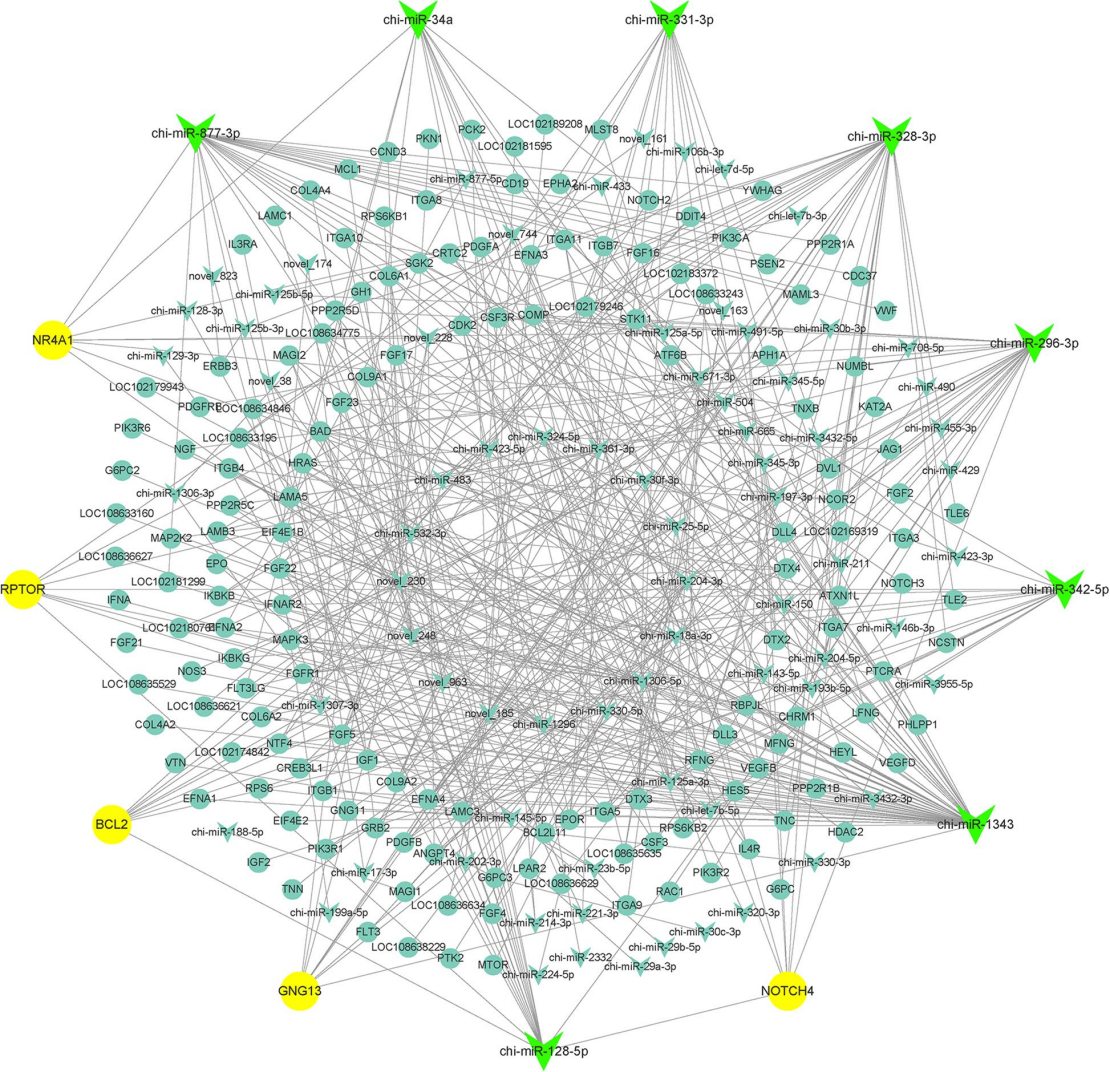
Protein–protein interaction network and network analysis for core genes and miRNAs

Based on the GO and KEGG target gene annotation, a target regulatory network consisting of 85 differentially expressed miRNAs and 168 genes related to ovary development was constructed using Cytoscape software (Fig. 8). Based on the degree of interaction, the network contained 8 core miRNAs and 5 core genes. The 8 core miRNAs were chi-miR-1343, chi-miR-328-3p, chi-miR-877-3p, chi-miR-296-3p, chi-miR-128-5p, chi-miR-331-3p, chi-miR-342-5p and chi-miR-34a. Similarly, the 5 core genes are BCL2, GNG13, NOTCH4, NR4A1, and RPTOR. chi-miR-1343, chi-miR-328-3p, chi-miR-877-3p and chi-miR-296-3p had 23 or more regulatory target genes. Among them, chi-miR-1343 had the highest number of target genes, which amounted to 54 and had a particularly noteworthy role in miRNA-mediated regulation. According to bioinformatics analysis, chi-miR-328-3p, chi-miR-296-3p, chi-miR-128-5p, and chi-miR-342-5p can target and regulate the core genes RPTOR, chi-miR-328-3p, chi-miR-296-3p, chi-miR-128-5p, and chi-miR-34a can target and regulate the core genes NOTCH4, and chi-miR-128-5p and chi-miR-34a can also target NR4A1 and BCL2, which may be involved in or affect the regulation of gonadal development, ovarian steroidogenesis, and follicular maturation.

### Experimental validation

**Fig. 9:**
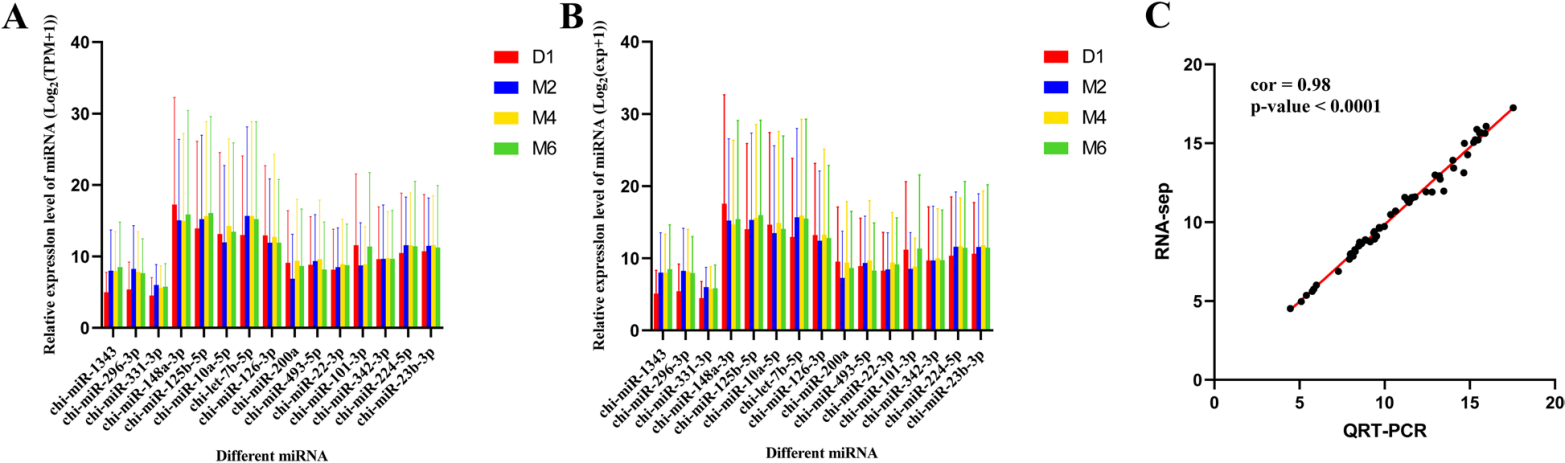
Comparison of relative expression of DE miRNA s in RNA-Seq and qRT-PCR results. **A** shows the results of RNA-seq. **B** shows the results of qRT-PCR. **C** Correlation between RNA-seq and qRT-PCR data

The qRT‒PCR results (Fig. 9A, B and Supplementary Table 10) proved that the expression patterns of the 15 randomly selected miRNAs were consistent with the Illumina sequencing results. We found that the expression levels of the 15 randomly selected miRNAs varied considerably, and to better present the trend of miRNA changes, we performed log2(exp + 1) transformation of the expression levels and correlation analysis between the RNA-seq data and the qRT‒PCR data (Fig. 9C) and found that the correlation (cor) value was 0.98 (*P* < 0.0001). This result confirmed the reliability of the sequencing results.

## Discussion

To study the serum levels of reproductive hormones during sexual maturation in goats, we measured the levels of FSH, LH, E2 and PROG. Puberty is a critical stage of life in the physiological changes associated with sexual maturation, representing a complex process subject to multiple endocrine and genetic controls [38]. Pubertal onset is considered to begin with an increase in GnRH, which activates the HPO axis [39]. Hypothalamic activation and secretion of GnRH are key upstream regulators that initiate the reproductive cascade to stimulate pulsatile release of FSH and LH [40]. During prepubertal stages, the levels of LH, FSH and E2 have been reported to increase gradually [41]. LH and FSH stimulate the secretion of steroid hormones in the gonads, while high levels of E2 exert negative feedback effect on the hypothalamus and anterior pituitary, thereby reducing the secretion of gonadotropins [42]. From mid-puberty onwards, E2 also produces positive feedback, which is a necessary process for ovulation. This requires sufficient LH release from the pituitary and for the follicles to reach a sufficient size to produce enough E2. Oestradiol increases pituitary sensitivity to GnRH, thereby increasing LH secretion [43]. The rise in E2 inhibits the secretion of FSH within the ovaries, causing the dominant follicle to undergo luteinization under the influence of LH, followed by ovulation. In the present study, we observed that the average levels of LH and FSH increased with the developmental process of puberty, but the rise in LH was more substantial than that of FSH. These elevations may be a result of higher baseline levels of both LH and FSH, as well as an increase in the frequency of LH surges [44]. Under the influence of high levels of E2, the levels of FSH and LH may exhibit a declining trend at the M6 stage. In addition, exogenous PROG has been demonstrated to increase gonadotropin levels [45]. PROG secretion at early prepuberty is regulated and influenced by LH, FSH and E2 [46]. We observed that at the M2 stage, PROG levels were significantly higher than those at the D1 stage, possibly because of the cooperative promotion of follicle development and ovulation by PROG and E2 and subsequent ovarian luteinization and an increase in PROG levels [47]. A delicate balance between different reproductive hormone signals is needed for proper development during sexual maturation.

The main function of the ovary is to affect the oestrous cycle and fertility of mammals, making it an important reproductive organ [48]. miRNAs are important regulators of gene expression and play key roles in ovarian disease prevention, ageing, cell proliferation, apoptosis and metabolism [49]. Many miRNAs have been identified from ovarian tissues or germ cells of goats [50–52], sheep [53–55], cows [56, 57], and humans [58, 59]; however, a complete analysis of the transcriptome of intact ovaries during the sexual maturity period of goats, especially Jining Grey goats, has rarely been reported thus far. Our analysis of the four developmental stage libraries identified a total of 667 miRNAs; only 474 miRNAs were commonly expressed in all four stages (Fig. 2A), suggesting that the expression of many miRNAs is time-specific [60]. The predominant length of the miRNAs we identified was 22 nt (Fig. 2B, C). This finding is consistent with previous findings in sheep [61], goats [1], pigs [62], and mice [63] but differed in bovine ovaries, where miRNAs of 20 nt size were most abundant [64], which may reflect species differences. According to the family analysis, the let-7 family had the highest number of annotated miRNAs (Fig. 2E). The family is one of the earliest miRNA groups discovered, and its family members are highly conserved throughout the species [65]. The let-7 family plays an important role in a variety of biological processes, such as cell proliferation, differentiation, tissue development, and tumour suppression [66]. Studies have shown that the expression of let-7 family members varies during follicular atresia. For example, downregulation of let-7c is associated with premature ovarian failure, suggesting that let-7c plays a key role in promoting healthy follicular development. Interestingly, the expression pattern of let-7 g in follicles appears to be different from that of other members of the family, as it is significantly upregulated during the atresia period [67]. The antiapoptotic gene MAP3K1 (mitogen-activated protein kinase kinase kinase 1) was found to be a direct target of let-7 g. Downregulation of MAP3K1 by let-7 g leads to the expression and dephosphorylation of the transcription factor FoxO1, which accumulates in the nucleus and triggers granulosa cell apoptosis [68]. These data suggest that the let-7 miRNA family has great potential in regulating follicular atresia, but further studies are needed to fully elucidate its underlying mechanisms.

Gene expression patterns can be inferred from differences in expression levels [69]. In this study, we analysed the expression levels of miRNAs and found 254 differentially expressed miRNAs across the four developmental stages. Among them, the majority of miRNAs exhibited differential expression exclusively in the D1 vs. M2, D1 vs. M4, and D1 vs. M6 comparison groups, with the number of DE miRNAs exceeding 170 in each of these groups. Therefore, we hypothesized that the period between D1 and M2 is a critical period for ovarian development, whereas the expression patterns of miRNAs and their main functions were similar in stages M2, M4 and M6, which may be due to these miRNAs stabilizing follicular development and maturation in goats after the M2 stage. We identified 15 miRNAs with high expression levels at all stages (Fig. 4). miR-148a had the highest average expression level and was reported to be the most highly expressed miRNA in follicular fluid extracellular vesicles of large and small follicles in goats [70]. miR-10a plays a key role in inducing apoptosis and inhibiting cell proliferation in ovarian GCs by inhibiting the brain-derived neurotrophic factor (BDNF) and TGF-β pathways [71]. In addition, miR-10a also regulates lipid metabolism and steroid hormone synthesis in sheep GCs by targeting the PTEN-AKT/WNT pathway [72]. Let-7a-5p, let-7c-5p, let-7b-5p, and let-7e-5p belong to the conserved let-7 family. As key regulators of gene expression, let-7 miRNAs are highly expressed in mammalian gonadal tissues [67, 73], and have been shown to regulate sperm formation and oocyte maturation [74, 75], as well as participate in the initial regulation of mammalian sexual maturation [76–78]. This study showed that the expression levels of let-7a/b were significantly higher than those of other let-7 miRNAs in tissues associated with ovarian development, suggesting that let-7a/b may play a more important role in ovarian development, which is in line with reports that a large number of miRNAs have been identified in mammalian ovaries. This is consistent with previous findings that let-7a and let-7b are two of the ten most abundant miRNAs in human, cow, and sheep ovaries [79–81]. These findings suggest that different members of let-7 miRNAs may have distinct roles during animal ovarian development and that their expression is stage dependent. Moreover, miR-125 is produced from the polycistronic let-7 complex gene region, and its expression disrupts the Notch-activated positive feedback loop, shifting the cell from receiving Notch signals to sending Notch signals [82]. Angiogenesis plays a crucial role in ovarian development and the restoration of ovarian function [83]. miR-126-3p has been shown to be important for vascular integrity and angiogenesis [84, 85].

To investigate the functions of DE miRNAs with distinct expression profiles, we analysed the expression patterns of DE miRNAs and identified three clusters (Fig. 5). The results revealed that the expression profiles of DE miRNAs were correlated with the developmental stages of goats. The miRNAs in Cluster 1 were upregulated from stage D1 to stage M2, with higher expression levels in the M2 group. Their target genes were significantly enriched in the MAPK signaling pathway, calcium signaling pathway, Ras signaling pathway, oestrogen signaling pathway, and glycerophospholipid metabolism. An increase in Ca2 + /CaMKII may activate the ERK and MAPK pathways in cumulus cells, promoting amphiregulin and epiregulin expression as well as progesterone production and, thus influencing cell cycle progression [86]. EGF induces CREB activity via the MAPK3/1 and Ca^2+^/CaMKII signaling pathways, promoting the expression of expansion-related genes and the expansion of cumulus cells [87]. Impairment of FGF23 function prior to primordial follicle formation leads to activation of the MAPK signaling pathway in mouse oocytes, which induces massive apoptosis of oocytes in the ovary. Previous studies have shown that the above signaling pathway may be involved in gonadal development, ovarian steroidogenesis and oocyte maturation [88]. The DE miRNAs in Cluster 2 were most highly expressed in the D1 group, and their target genes were mainly enriched in signaling pathways related to cellular processes, such as metabolic pathways, neutrophil extracellular trap formation, amino sugar and nucleotide sugar metabolism, and folate biosynthesis, with the highest number of enriched genes being metabolic pathways. Rapid morphological growth of the ovary and a significant increase in ovarian weight and volume (5 times higher than at birth) have been reported in goats during 0–30 days after birth [2]. This is consistent with our experimental results that the expression of genes related to organ and tissue growth and metabolic processes is higher in the D1 group after goat birth in goats. Cluster 3 showed that the expression levels of DE miRNAs increased from the D1 to M4 stages, with higher expression levels in the M4 group. Their target genes were significantly enriched in the RIG-I-like receptor signaling pathway, MAPK signaling pathway, PI3K-Akt signaling pathway, Notch signaling pathway and Rap1 signaling pathway, as well as other metabolic-related pathways. The PI3k-Akt signaling pathway induces the transcription of target genes and mediates angiogenesis, cell invasion, metastasis, proliferation and apoptosis [89]. During follicle development, the PI3K signaling pathway controls primordial follicle activation through FOXO3. During the assembly of primordial follicles, FOXO3 is nonphosphorylated and localized in the nucleus, where it acts as an inhibitor of primordial follicle activation. Activation of PI3K/AKT leads to the phosphorylation of FOXO3 and its export from the nucleus, which triggers the activation of primordial follicles [90]. The Notch signaling pathway is an evolutionarily conserved pathway that plays a major role in cell proliferation, differentiation, migration, adhesion, and apoptosis, among many other cellular processes. It has been shown that constitutive Notch signaling in adult transgenic mice inhibits bFGF-induced angiogenesis and follicular development [91] and that granulosa cell proliferation is also dependent on Notch signaling [92]. The Rap1 signaling pathway regulates cell adhesion, cell‒cell junction formation, and cell polarity by cycling between inactive GDP-bound and active GTP-bound conformations [93]. Therefore, our experimental results suggest that in the early postnatal period of Jining Grey goats, ovarian development may be related to cell growth, which is mainly manifested by the rapid growth of ovarian morphology. During sexual maturation, ovarian function and performance were enhanced, as evidenced by the development and maturation of follicles and ovaries. This is similar to the observations reported by Shi [2]. Overall, spatially and temporally specific expression of miRNAs in the ovaries of Jining Grey goats plays a critical role in ovarian development and early maturation.

We constructed a regulatory network of miRNAs and target genes associated with ovarian development. Chi-miR-1343 had the highest number of target genes, and its targets were significantly enriched in the PI3K-Akt signaling pathway. miR-1343 has been reported to play an important functional role in bovine follicular development, as its expression level increases during the transition from secondary follicles to preovulation [94]. Moreover, TGFBR1, a type I receptor in the TGF-β signaling pathway, impedes GC apoptosis of GCs in the ovary, and miR-1343 silences the expression of TGFBR1. Interestingly, miR-1343 was found to downregulate TGFBR1 expression, which promotes the apoptosis pathway and inhibits cell proliferation [95]. Our results showed that miR-1343 was expressed at a lower level in the D1 group and at higher levels in the M2, M4 and M6 groups, which further indicates that its function may be related to the maintenance of ovarian physiological function, but more experimental evidence is still needed. miR-331-3p is a member of the miR-331 family and has been shown to be a tumour suppressor miRNA [96]. It has been reported that miR-331-3p targets HER2 through the PI3K/Akt and extracellular signal-regulated protein kinase 1 and 2 (ERK1/2) pathways, thereby inhibiting the proliferation of colorectal cancer cells [97]. In gastric cancer, miR-331-3p directly targeted E2F1 and induced cell growth defects [98]. It was found that miR-331-3p could inhibit tumour cell invasion and metastasis by regulating ErbB2 and VAV2 to attenuate the epithelial–mesenchymal transition in non-small cell lung cancer [99]. These findings indicate that miR-331-3p plays an important role in tumour suppression during cancer development. However, the role of miR-331-3p in ovarian development is still unclear and requires further research and exploration. miR-342-5p is an intron hosted in the Ena/Vasodilator-Stimulated Phosphoprotein-Like (Ena/VASP-like, EVL) gene miRNA, belonging to the Ena/VASP family, is involved in actin cytoskeleton remodelling and has been reported to enhance ERK-maintained cell proliferation [100]. As a downstream effector of Notch signaling, miR-342-5p regulates the proliferation and differentiation of mouse neural stem cells [101]. It was previously reported that miR-296-3p is expressed in mouse ovaries [102], inhibits cell plasticity in different tumour lines [103], and promotes apoptosis in liver [104] and mammalian pancreatic α cells [105]. Furthermore, miR-296 was observed to be epigenetically regulated as part of the imprinted Gnas/GNAS clusters [106]. miR-296-3p, miR-328-3p, miR-128-5p and miR-34a all target NOTCH4, which was significantly enriched in the Notch signaling pathway. The Notch pathway comprises a conserved family of transmembrane receptors that interact with a number of specific ligands to regulate cell fate [107]. Notch signaling plays a key role in many developmental processes, affecting cellular differentiation, proliferation, and apoptosis [108, 109]. In addition, NOTCH4 has been identified as a novel factor involved in the regulation of angiogenesis [110]. Overall, we predicted that core miRNAs and potential target genes may be involved in the regulation of ovarian development.

## Conclusion

The present study investigated the changes in serum reproductive hormone levels during the sexual maturity period in goats. The serum levels of FSH, LH, OT, PRL, E2, and PROG gradually increased in goats, reaching peak secretion at four months of age. The results of small-RNA sequencing revealed that there were 254 differentially expressed miRNAs and three miRNA clusters with unique expression patterns during the four developmental stages of sexual maturity in Jining Grey goats. In addition, a regulatory network of miRNAs involved in ovarian development was constructed. Functional enrichment analysis showed that the target genes were mainly concentrated in pathways related to cell proliferation, cell differentiation, hormone secretion and metabolism. Our results provide a more comprehensive description of the hormonal changes during the sexual maturity period in goats and point to the differentially expressed miRNAs in the ovary that may be associated with sexual maturation. Our study provides valuable insights into ovarian development during the sexual maturity period in goats from both endocrine and genetic perspectives.

### Supplementary Information

**Additional file 1: Supplementary Table 1.** Weight and ovarian-related indicators of Jining grey goats for miRNA profile analysis.

**Additional file 2: Supplementary Table 2.** The RT-qPCR primers used for validation.

**Additional file 3: Supplementary Table 3.** Clean reads filtering results.

**Additional file 4: Supplementary Table 4.** ncRNA and repeat sequence annotation.

**Additional file 5: Supplementary Table 5.** Summary of sequencing read alignment to the goat reference genome.

**Additional file 6: Supplementary Table 6.**

**Additional file 7: Supplementary Table 7.**

**Additional file 8: Supplementary Table 8.**

**Additional file 9: Supplementary Table 9.**

**Additional file 10:** **Supplementary Table 10.** Expression fold change and *p*-value of DE miRNAs in RNA-Seq.

## Data Availability

The datasets used and/or analysed during the current study are available from the corresponding author on reasonable request. The sequencing data were submitted to the Sequence Read Archive (Accession Numbers PRJNA1011805) in NCBI.

## Abbreviations

FSH: Follicle stimulating hormone
LH: Luteinizing hormone
miRNA: MicroRNA
DEMs: Differentially expressed MicroRNA
BP: Biological processes
CC: Cellular components
MF: Molecular functions
GO: Gene ontology
KEGG: Kyoto Encyclopedia of Genes and Genomes
